# IFIT3 Is Increased in Serum from Patients with Chronic Hepatitis B Virus (HBV) Infection and Promotes the Anti-HBV Effect of Interferon Alpha via JAK-STAT2 *In Vitro*

**DOI:** 10.1128/spectrum.01557-22

**Published:** 2022-10-31

**Authors:** Siyi Xu, Jinlan Huang, Zhen Xun, Shiqi Li, Ya Fu, Ni Lin, Wennan Wu, Tianbin Chen, Can Liu, Qishui Ou

**Affiliations:** a Department of Laboratory Medicine, Gene Diagnosis Research Center, the First Affiliated Hospital, Fujian Medical University, Fuzhou, China; b Fujian Key Laboratory of Laboratory Medicine, the First Affiliated Hospital, Fujian Medical University, Fuzhou, China; c First Clinical College, Fujian Medical University, Fuzhou, China; State Key Laboratory of Microbial Resources, Institute of Microbiology, Chinese Academy of Sciences

**Keywords:** IFIT3, chronic hepatitis B, interferon

## Abstract

Increasing evidence indicates that interferon alpha (IFN-α) therapy is an effective treatment option for a subgroup of patients with chronic hepatitis B virus (HBV) infection. It has been confirmed that interferon-induced protein with tetratricopeptide repeats 3 (IFIT3), a member of the interferon-stimulated genes (ISGs), could inhibit the replication of various viruses. However, its effect on HBV replication is unclear. The present study sought to explore the role and mechanism of IFIT3 in IFN-α antiviral activities against HBV. IFIT3 mRNA levels in the peripheral blood of 108 treatment-naive patients and 70 healthy controls were analyzed first. The effect of IFIT3 on the Janus kinase-signal transducer and activator of transcription (JAK-STAT) signaling pathway under the dual intervention of IFN-α and HBV was also explored *in vitro*. Treatment-naive individuals exhibited elevated levels of IFIT3 mRNA compared to the controls (*P* < 0.0001). Mechanistically, the knockdown of IFIT3 inhibited the phosphorylation of signal transducer and activator of transcription 2 (STAT2), whereas the overexpression of IFIT3 produced the opposite effect *in vitro*. Meanwhile, the overexpression of IFIT3 enhanced the expression of IFN-α-triggered ISGs, including myxovirus resistance A (MxA), 2′-5′-oligoadenylate synthetase 1 (OAS1), and double-stranded RNA-activated protein kinase (PKR), while a weaker induction of IFN-α-triggered ISGs was observed in ruxolitinib-treated cells. After decreasing IFIT3 expression by validated small hairpin RNAs (shRNAs), the levels of hepatitis B surface antigen (HBsAg), hepatitis B e antigen (HBeAg), and HBV DNA secreted by HepG2 cells transiently transfected with the pHBV1.2 plasmid were increased. Our findings suggest that IFIT3 works in a STAT2-dependent manner to promote the antiviral effect of IFN-α through the JAK-STAT pathway in HBV infection in both human hepatocytes and hepatocarcinoma cells.

**IMPORTANCE** Our study contributes new insights into the understanding of the functions and roles of interferon-induced protein with tetratricopeptide repeats 3 (IFIT3), which is one of the interferon-stimulated genes induced by hepatitis B virus infection in human hepatocytes and hepatocarcinoma cells, and may help to identify targeted genes promoting the efficacy of interferon alpha.

## INTRODUCTION

Hepatitis B virus (HBV) infection is prevalent in the world and is regionally different. There are approximately 257 million chronic HBV (CHB) infections worldwide, accounting for 3.5% of the global population (1). According to statistics, more than 70% of the onset of liver cirrhosis and hepatocellular carcinoma (HCC) is related to HBV infection (2).

Pegylated interferon alpha (Peg-IFN-α) is a type I interferon (IFN-I), which mainly induces changes in the expression of interferon-stimulated genes (ISGs) by activating the Janus kinase-signal transducer and activator of transcription (JAK-STAT) pathway and produces effector products that play an important antiviral role in different stages of the virus life cycle (3). In recent years, ISGs such as apolipoprotein B mRNA-editing enzyme catalytic polypeptide 3B (APOBEC3G) (4), tripartite motif-containing 25 (TRIM25) (5), myxovirus resistance A/B (MxA/B) (6), and TRIM14 (7) induced by Peg-IFN-α have been reported to inhibit HBV replication (4–7). IFN-α not only exhibits direct inhibition of HBV DNA replication but also clears infected hepatocytes through the indirect regulation of host immunity (8). However, Peg-IFN-α was evidenced to be effective for only 20% to 40% of CHB patients (9). There are pronounced individual differences after the clinical use of Peg-IFN-α treatment, which may be because induced ISGs can inhibit only viral replication and are not enough to eliminate the virus (10), or efficacy depends on the balance between the antiviral effect of Peg-IFN-α and the antagonistic effect of host factors (11). Recently, a consensus definition for “complete cure” has been proposed as the treatment endpoint for new HBV therapies, which was described as a functional cure plus covalently closed circular DNA (cccDNA) elimination (12). This is partly due to the viral replication strategy of HBV. After infection of hepatocytes, HBV DNA is transported to the nucleus and converted into a minichromosome (cccDNA), and persistent or residual cccDNA resides as an episomal template in the nuclei of infected hepatocytes (13). IFN-α has demonstrated the ability to reduce the levels of HBV DNA, hepatitis B surface antigen (HBsAg), and cccDNA efficiently (14) not only through a direct mechanism (cccDNA degradation or epigenetic silencing) but also by exerting robust immunoregulatory functions (15). Therefore, the specific and efficient activation of the IFN-α pathway in HBV patients might represent one treatment strategy to eradicate HBV from the infected liver. However, how to enhance the efficacy of IFN-α is a problem worthy of discussion.

The interferon-induced protein with tetratricopeptide repeats (IFIT) family with multiple triangular tetrapeptide repeats has been favored by researchers in related antivirus fields (16–18). Humans have a unique combination of IFIT family genes, including IFIT1 (ISG56), IFIT2 (ISG54), IFIT3 (ISG60), and IFIT5 (ISG58), among which IFIT3 is widely expressed in the human liver (19). The promoter regions of IFITs contain two or three IFN-stimulated gene regulatory elements (ISREs), which can be strongly induced by IFN (mainly IFN-I), viral infections, and lipopolysaccharides and play an important role in the antiviral response (18). Among the IFITs, most of the existing literature focuses on IFIT1 and IFIT2; only a few studies picked IFIT3 as the main gene, in which it was found that IFIT3 activation can inhibit porcine reproductive and respiratory syndrome virus (PRRSV) replication (20), vesicular stomatitis virus (VSV) (21), and dengue fever virus (DV) (22) through *in vitro* experiments. Interferon regulatory factor 3 (IRF3) and NF-κB activated by peroxisomal mitochondrial antiviral signaling (MAVS) can induce the production of IFIT3 directly, which then promotes the interaction between TNFR-associated factor family member-associated NF-kB activator-binding kinase 1 (TBK1) and mitochondrial MAVS, further amplifying antiviral signaling (23). However, when focusing on the cross talk between IFIT3 and HBV infection, just one study reported that IFIT3 could be induced by HBx in an NF-κB-dependent manner, leading to enhanced HBV replication (24), while another study suggested that IFIT3 promotes IFN-α effector responses and therapeutic effects on HCC (25). Since HBV infection is closely related to the onset of HCC, further research will be needed to fully understand the mechanisms of IFIT3’s function in anti-HBV activity.

Here, we investigated the effects of IFIT3 on IFN-α anti-HBV replication and further demonstrated the crucial role of signal transducer and activator of transcription 2 (STAT2) in regulating IFN-α-induced IFIT3 expression. This study provides new insights into the understanding of the functions and roles of IFIT3, which is one of the ISGs induced by HBV infection in human hepatocytes, and may help to identify targeted genes promoting the efficacy of IFN-α.

## RESULTS

### Differential expression of IFIT3 mRNA in the blood of treatment-naive HBV-infected patients and healthy controls.

**FIG 1:**
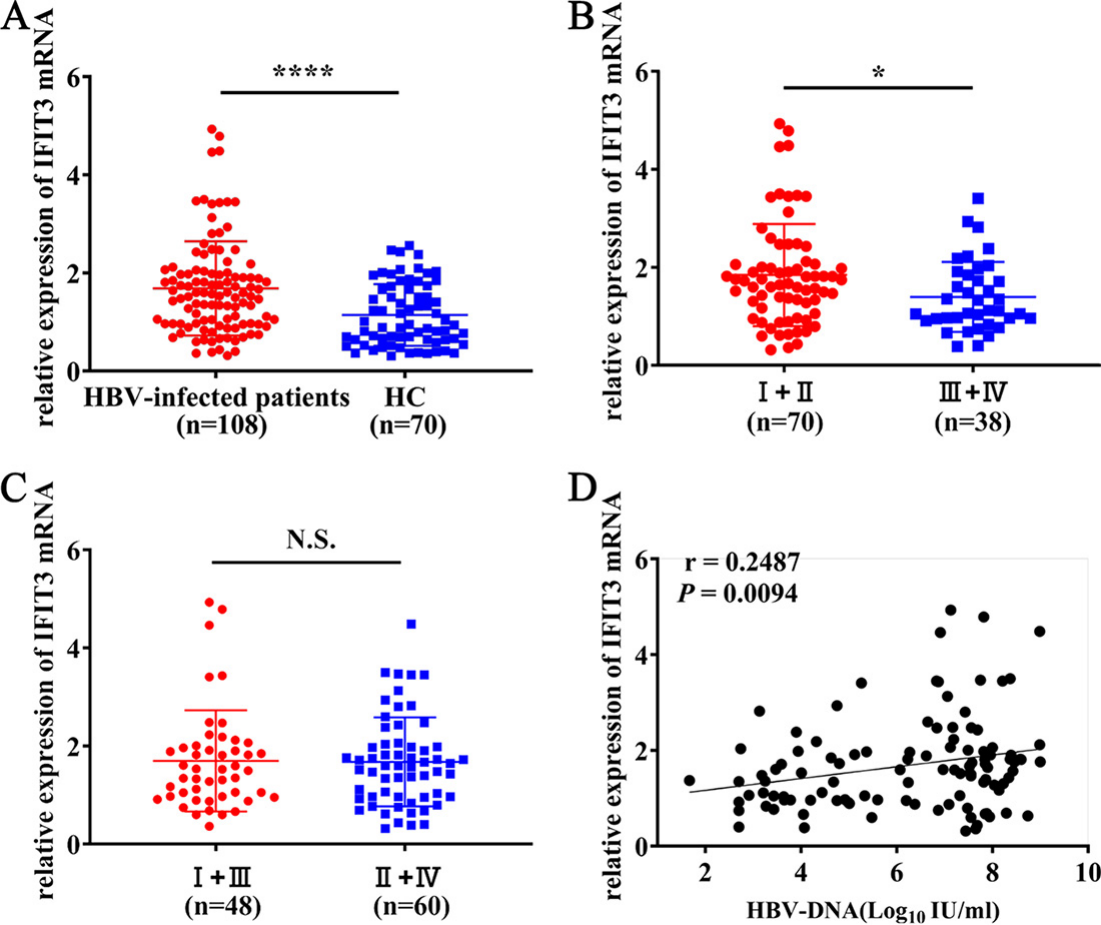
IFIT3 mRNA expression in the blood of treatment-naive HBV-infected patients and healthy controls. (A to C) Relative expression levels of IFIT3 mRNA in treatment-naive HBV-infected patients and healthy controls (HC). (D) Correlation between HBV DNA levels and IFIT3 mRNA expression in treatment-naive HBV-infected patients. Serum levels of HBV DNA were detected by qRT-PCR. All data are presented as means ± SD. ****, *P *< 0.0001; *, *P *< 0.05; N.S., not significant. Group I, HBeAg-positive patients with chronic hepatitis; group II, HBeAg-positive patients with chronic infection; group III, HBeAg-negative patients with chronic hepatitis; group IV, HBeAg-negative patients with chronic infection.

IFIT1 and IFIT2 fully exert antiviral functions at baseline expression levels. To identify whether IFIT3 was associated with the process of HBV infection, peripheral blood samples from 108 treatment-naive HBV-infected patients and 70 healthy controls were collected to determine IFIT3 mRNA expression. As shown in Fig. 1A, the IFIT3 mRNA levels in the blood were significantly lower in the controls than in the treatment-naive HBV-infected patients (*P *< 0.0001). Patients who were hepatitis B e antigen positive (HBeAg^+^) (group I and group II) had significantly higher IFIT3 mRNA expression levels than did those who were HBeAg negative (HBeAg^−^) (group III and group IV) (*P *= 0.0200) (Fig. 1B). IFIT3 mRNA expression in patients with or without elevated alanine transaminase (ALT) levels was analyzed, and no significant relationship was found between the groups with elevated ALT levels (group I and group III) and those with normal ALT levels (group II and group IV) (*P *= 0.9126) (Fig. 1C). Furthermore, the baseline levels of HBV DNA showed a positive correlation with IFIT3 mRNA expression (*r* = 0.2485; *P *= 0.0094) (Fig. 1D). Taken together, these results showed that IFIT3 levels are significantly higher in HBeAg-positive patients. HBV infection enhanced the expression level of IFIT3 in the blood to a certain extent.

### Induction of IFIT3 expression by IFN-α and HBV in hepatocarcinoma cell lines and human hepatocytes.

**FIG 2:**
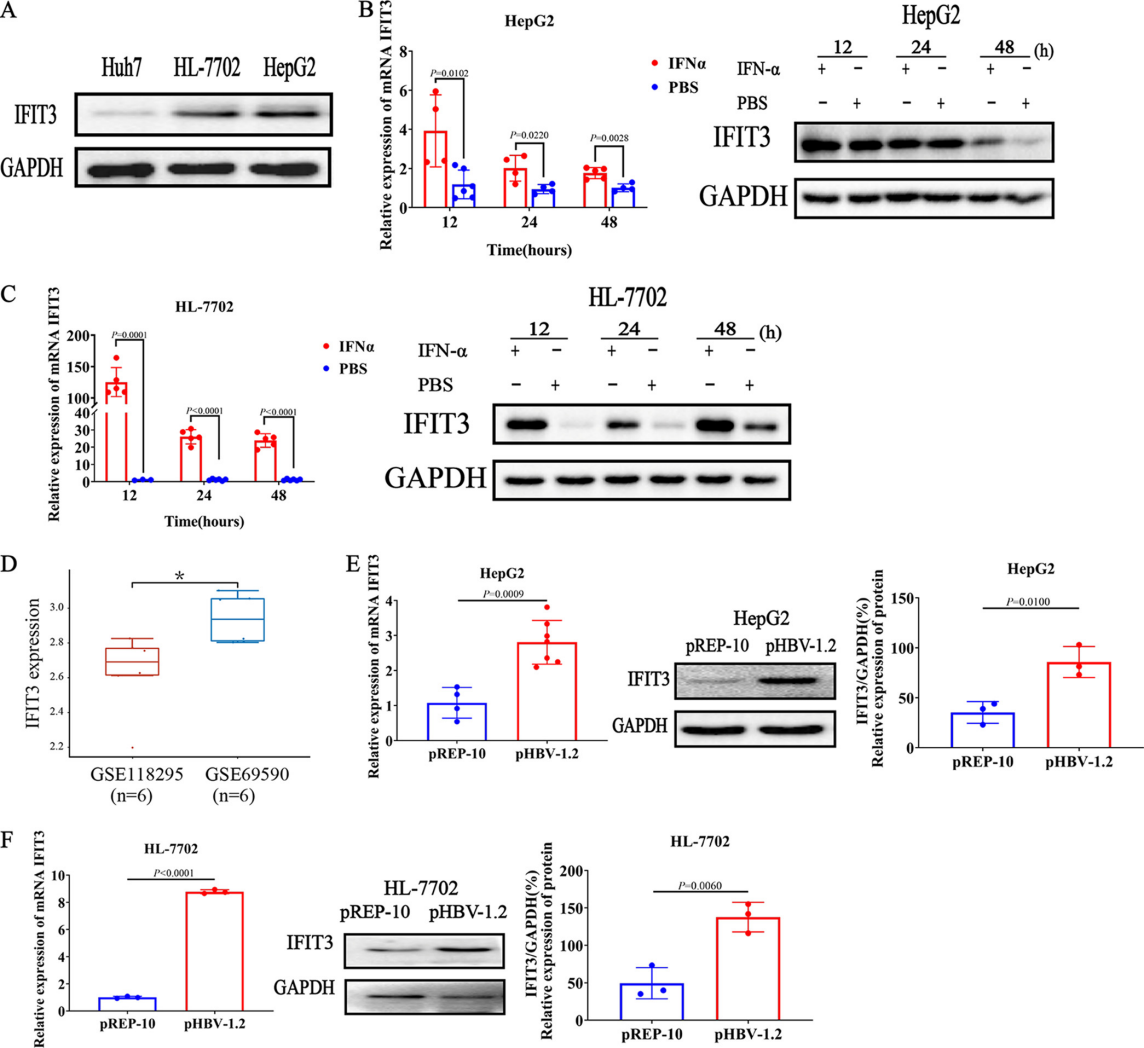
Induction of IFIT3 expression by exogenous IFN-α and HBV in HepG2 cells and HL-7702 cells. (A) Huh7, HL-7702, and HepG2 cells were seeded into a 6-well plate overnight, and the cells were then harvested. IFIT3 expression was detected by Western blotting. (B and C) HepG2 (B) and HL-7702 (C) cells were cultured in 12-well plates and treated with type I IFN-α (1,000 IU/mL) or PBS for the indicated times (12, 24, or 48 h). (D) Distribution of the expression of the IFIT3 gene in tissues. The horizontal axis represents different groups of samples, and the vertical axis represents the distribution of gene expression. (E and F) HepG2 cells (E) and HL-7702 cells (F) were transiently transfected with pHBV-1.2 or pREP-10 (empty vector) for 48 h. The level of IFIT3 was analyzed by qRT-PCR and Western blot analysis. GAPDH is presented as the loading control. Western blot strips were quantified by densitometric analysis, and values in the bar graphs are shown as fold changes compared to the controls. GSE118295, GEO accession no. for primary human hepatocytes (PHHs) without HBV stimulation; GSE69590, GEO accession no. for PHHs with HBV stimulation. *, *P *< 0.05. Data are representative of results from three independent experiments and are presented as means ± SD (*n* = 3).

To investigate the level of IFIT3 in liver cells, the endogenous expression of IFIT3 in hepatocarcinoma cell lines (Huh7 and HepG2) and a human hepatocyte cell line (HL-7702) was evaluated. As shown in Fig. 2A, IFIT3 expression was detected in all three liver cell lines by Western blotting. HL-7702 and HepG2 cells were chosen for further experiments. IFN-α-induced IFIT3 expression in HepG2 and HL-7702 cells and the effect of IFIT3 on HBV replication were assessed. The results revealed that the expression of IFIT3 was constant in HepG2 cells and was upregulated by IFN-α stimulation (Fig. 2B). Furthermore, the induction of IFIT3 expression by IFN-α was more significant in HL-7702 hepatocytes than in HepG2 cells (Fig. 2C). The mechanisms of the regulation of the expression of IFIT3 were different between the two cell lines. The basal and cytokine-induced expression levels of IFIT3 indicate a potentially important role of IFIT3 in host defense mechanisms against pathogen infections in hepatocytes. To characterize gene induction by HBV, two transcript expression microarray data sets downloaded from the Gene Expression Omnibus (GEO) (accession no. GSE118295 and GSE69590) were analyzed. The results demonstrated that compared with the control group (primary human hepatocytes [PHHs] without HBV infection), the induction of IFIT3 was detected after stimulation by HBV (*P *= 0.0150) (Fig. 2D). To further determine the effect of HBV on IFIT3, HepG2 and HL-7702 cells were transfected with the HBV1.2 plasmid (pHBV-1.2) viral genome construct. The expression of IFIT3 was significantly increased in HepG2 cells (Fig. 2E) and HL-7702 cells (Fig. 2F) following HBV infection. These results suggest that IFIT3 expression is positive to HBV infection in both hepatocarcinoma cell lines and human hepatocytes.

### IFIT3 modulates the expression of multiple genes involved in the HBV-related IFN-α-induced immune response *in vitro*.

**FIG 3:**
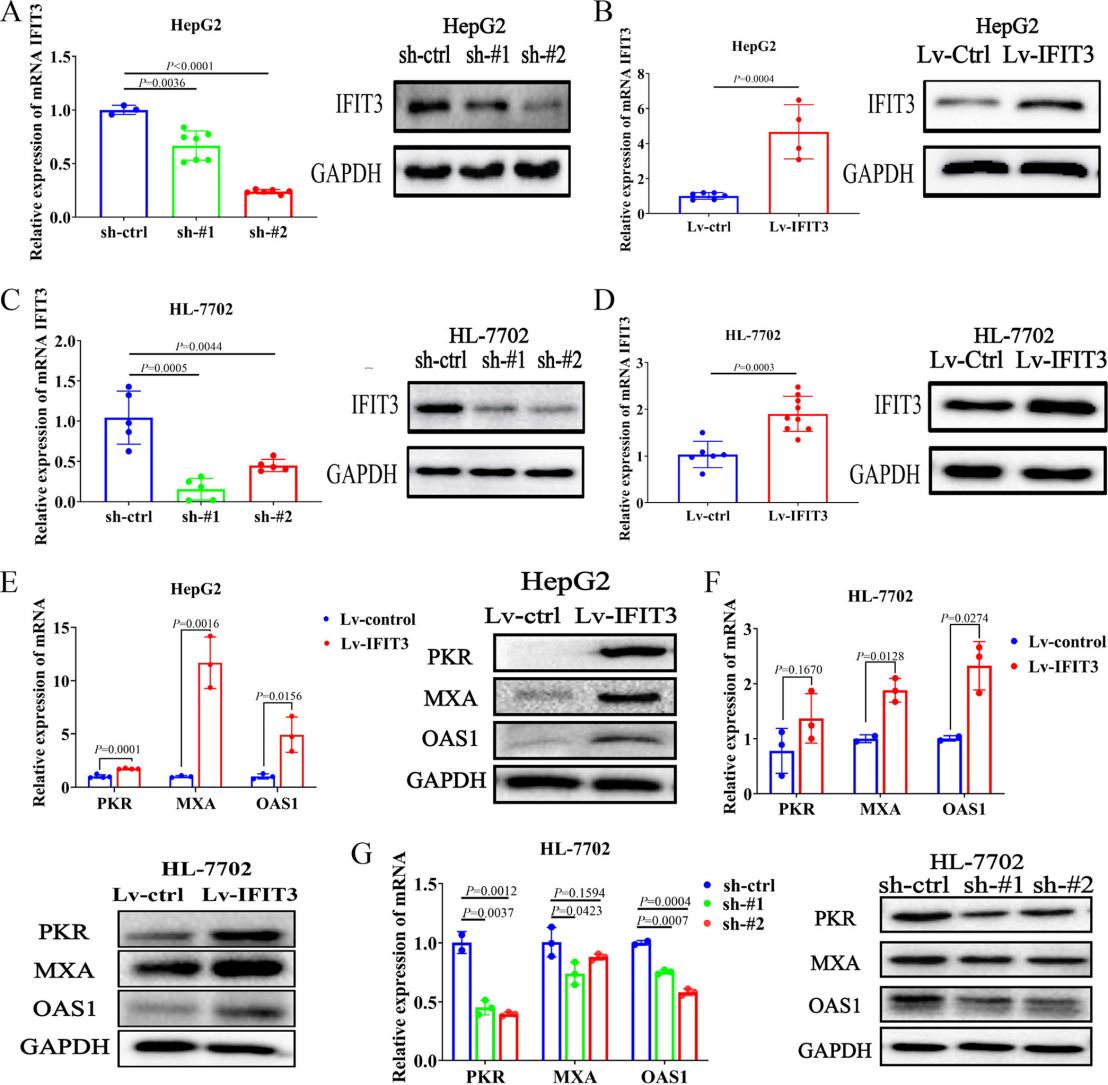
IFIT3 increases HBV-related IFN-α-induced ISGs *in vitro*. (A and C) Knockdown of endogenous IFIT3 in HepG2 and HL-7702 cells transduced with specific shRNAs (designated sh-control [sh-ctrl], sh-IFIT3#1 [sh-#1], and sh-IFIT3#2 [sh-#2]). GAPDH was used as a housekeeping gene for qRT-PCR and Western blot analysis. (B and D) HepG2 and HL-7702 cells were infected with lentiviruses carrying the IFIT3 gene (designated Lv-control [Lv-ctrl] or Lv-IFIT3). The level of IFIT3 was significantly increased in HepG2 and HL-7702 cells overexpressing IFIT3 compared with the control cells. GAPDH was used as a housekeeping gene for qRT-PCR and Western blot analysis. (E and F) HepG2 (E) and HL-7702 (F) cells stably overexpressing IFIT3 and infected with lentivirus were seeded into 6-well plates overnight and treated with IFN-α (1,000 IU/mL) for 48 h before being harvested. Induced ISG expression was analyzed by qRT-PCR and Western blot analysis. (G) HL-7702 cells with stably suppressed endogenous IFIT3 expression were seeded into 6-well plates overnight and treated with IFN-α (1,000 IU/mL) for 48 h before being harvested. The expression of the indicated genes was analyzed by qRT-PCR and Western blot analysis. Data are representative of results from three independent experiments and are presented as means ± SD.

To gain insight into the biological role of IFIT3 in Peg-IFN-α anti-HBV replication, lentiviral small hairpin RNA (shRNA) vectors were used to specifically and stably knock down the endogenous expression of IFIT3 in HepG2 and HL-7702 cells. Transfection with shRNA constructs targeting IFIT3 (sh-IFIT3) reduced IFIT3 expression by approximately 65% compared with the controls (Fig. 3A and C). HepG2 and HL-7702 cells stably overexpressing IFIT3 were also constructed by lentivirus infection (Fig. 3B and D).

During HBV infection, the therapeutic effect of IFN-α is potentially dependent on the changes in the expression of hundreds of ISGs (25). Three conventional ISGs, double-stranded RNA-activated protein kinase (PKR), MxA, and 2′-5′-oligoadenylate synthetase 1 (OAS1), were used to explore the effect of IFIT3 on the IFN-α-induced immune response. The results showed that the overexpression of IFIT3 promoted the expression of these ISGs in the HepG2 and HL-7702 cell lines (Fig. 3E and F), whereas the knockdown of IFIT3 in HepG2 cells inhibited IFN-α-induced ISG expression (Fig. 3G). These data suggest that IFIT3 positively regulates the HBV-related IFN-α-induced immune response in HepG2 and HL-7702 cells.

### IFIT3 strengthens IFN-α effector signaling by promoting STAT2 phosphorylation.

**FIG 4:**
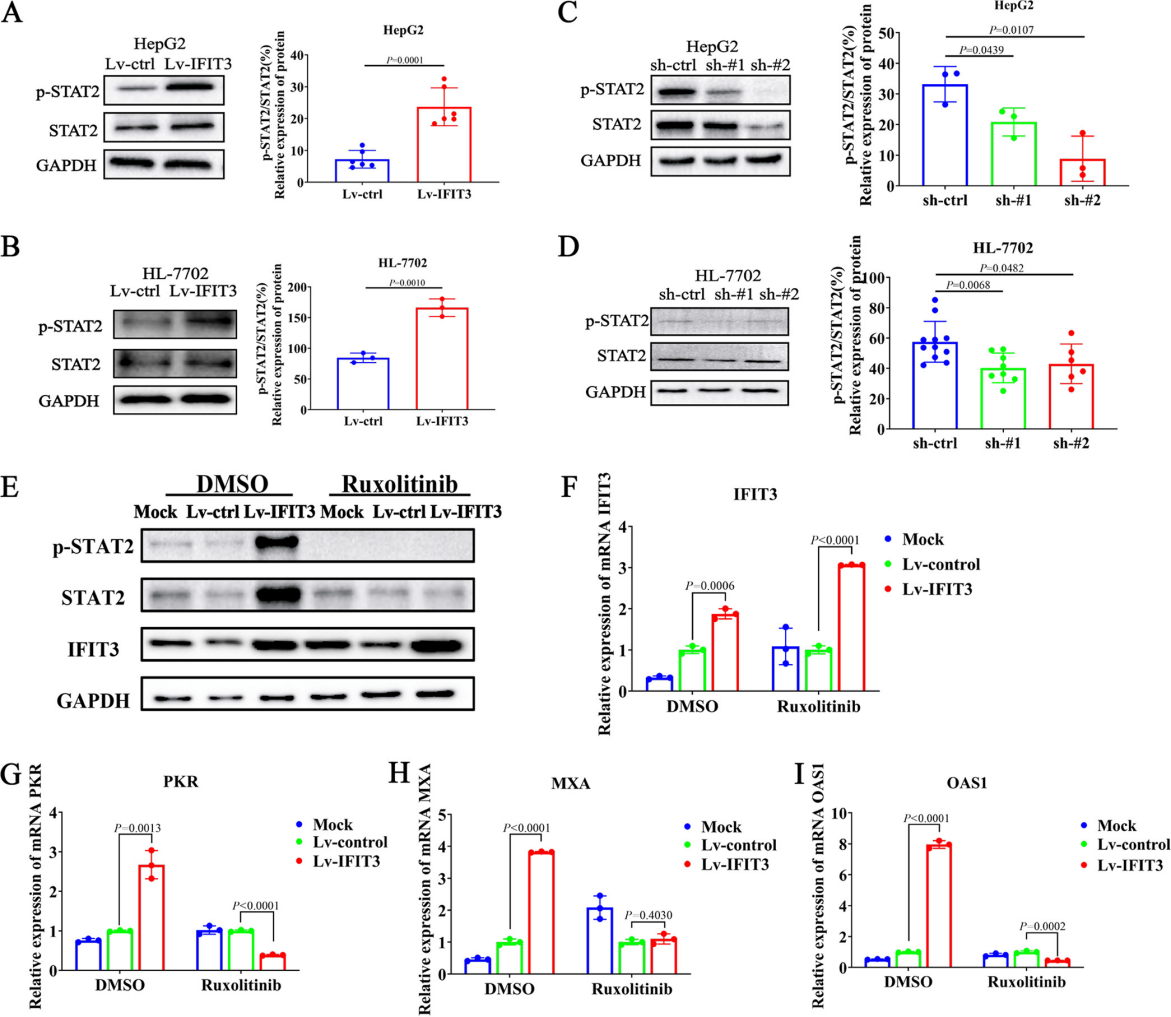
IFIT3 strengthens IFN-α effector signaling by promoting STAT2 phosphorylation. (A to D) HepG2 and HL-7702 cells with silenced or enhanced IFIT3 expression were seeded into 6-well plates overnight and then treated with IFN-α (1,000 IU/mL) for 30 min before being harvested. The levels of STAT2 and phosphorylated STAT2 (p-STAT2) were examined by Western blotting. GAPDH was used as an internal control. Western blot strips were quantified by densitometric analysis, and values in the bar graphs are shown as fold changes compared to the control. (E) HepG2 cells with enhanced IFIT3 expression were pretreated with ruxolitinib for 6 h before treatment with IFN-α (1,000 IU/mL) for 30 min. Cells were treated with dimethyl sulfoxide (DMSO), which served as a negative control. (F to I) HepG2 cells with enhanced IFIT3 expression were pretreated with ruxolitinib or DMSO for 6 h and then cultured with IFN-α (1,000 IU/mL) for 6 h. A control (without ruxolitinib or DMSO) was used for each treatment group. The experiments were performed in triplicate, and data are expressed as means ± SD.

To better understand the mechanism by which IFIT3 regulates the production of ISGs, the expression of several key regulators involved in these biological processes was assessed. Since IFN-α does not have antiviral activity, it binds to the interferon alpha/beta receptor (IFNAR) on the surface of target cells to activate the JAK-STAT signaling pathway, causing the phosphorylation of STAT1 and STAT2, and then induces the expression of ISGs (26, 27). Our results showed that the phosphorylation of STAT2 was increased in HepG2 and HL-7702 cells overexpressing IFIT3 (Fig. 4A and B) and decreased in cells with downregulated IFIT3 compared with the controls (Fig. 4C and D). The overexpression/knockdown of IFIT3 had little effect on the phosphorylation of STAT1 (data not shown).

Specific inhibitors of protein kinases serve as powerful tools to study select signaling pathways. To determine whether inhibiting the JAK-STAT pathway could reverse the promoted effects of IFIT3 on the expression of ISGs, cells were cultured with or without an inhibitor, ruxolitinib. Ruxolitinib was designed as a molecule with low-nanomolar potency selective for the JAK1 and -2 enzymes but without significant inhibition of non-JAK kinases. Notably, targeting JAK proteins successfully decreased the expression of phosphorylated STAT2 (p-STAT2) after treatment with ruxolitinib (Fig. 4E). Meanwhile, there was no influence on the expression of IFIT3 (Fig. 4F). Conversely, after treatment with ruxolitinib, the upregulation of ISGs (PKR, MxA, and OAS1) was suppressed compared with dimethyl sulfoxide (DMSO)-treated HepG2 cells (Fig. 4G to I). This observation was confirmed in one additional human hepatocyte cell line, namely, HL-7702. As shown in Fig. S1A and B in the supplemental material, the phosphorylation of STAT2 was inhibited after treatment with ruxolitinib, while the expression of IFIT3 was not affected. But the suppression effect of the upregulation of ISGs on hepatocytes was not obvious compared with that on hepatoma cells. Even so, the same trend was observed in HL-7702 cells (Fig. S1C to E). Therefore, our findings demonstrated a correlation between the suppression of STAT2 activation after JAK1 inhibition, evidenced by STAT phosphorylation, and the upregulation of ISGs through IFIT3 induction. IFIT3 plays an important role in the upregulation of ISGs by activating the JAK1-STAT2 pathway in HepG2 cells.

Together, these results suggest that IFIT3 can promote the phosphorylation of STAT2, thereby strengthening the IFN-α effector signaling pathway, leading to the more efficient production of ISGs.

### Overexpression of IFIT3 in HepG2 and HL-7702 cells significantly decreases HBsAg and HBeAg secretion and HBV replication.

**FIG 5:**
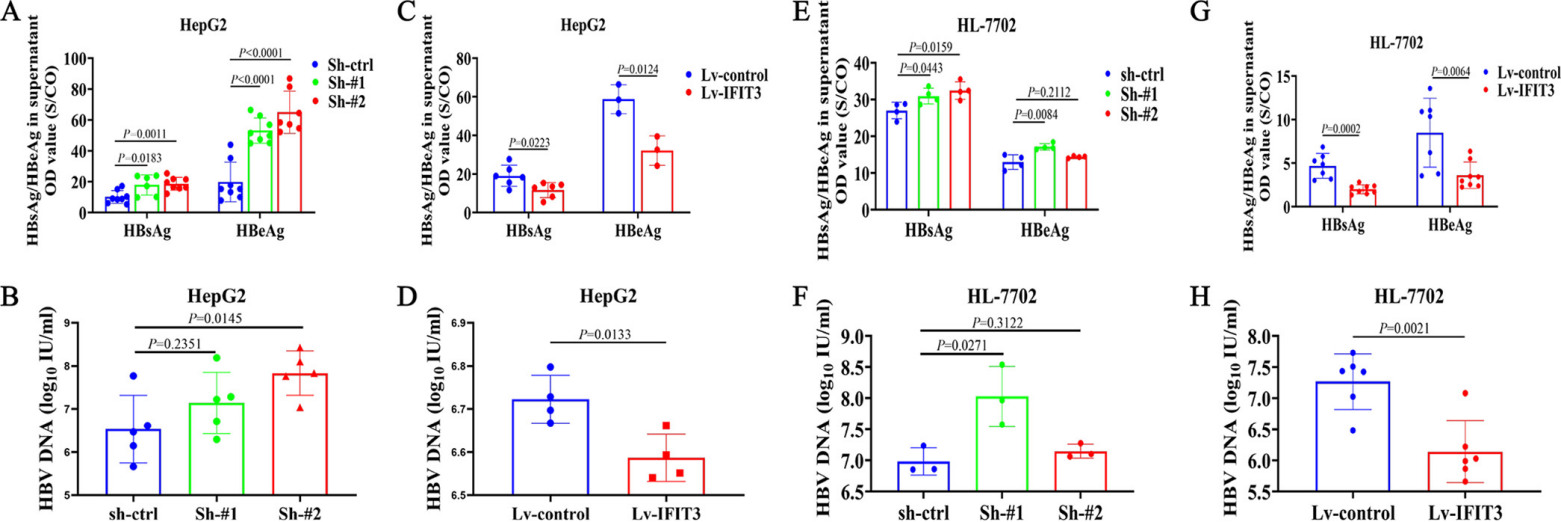
Overexpression of IFIT3 in HepG2 and HL-7702 cells significantly decreases HBsAg and HBeAg secretion and HBV replication. HepG2 or HL-7702 cells with enhanced or silenced IFIT3 expression were seeded into 6-well plates overnight. Transfection with pHBV-1.2 was performed the next day, followed by incubation for 72 h. (A, C, E, and G) HBsAg and HBeAg contents secreted into the supernatant were detected by a chemiluminescent-microparticle immunoassay. OD, optical density. (B, D, F, and H) HBV DNA was extracted and analyzed by qRT-PCR 3 days later.

To further validate the antiviral effect of IFIT3 on HBV replication, HepG2 cells were transfected with the pHBV-1.2 plasmid. As shown in Fig. 5A, the silencing of IFIT3 expression by validated shRNAs significantly increased HBsAg and HBeAg secretion in the culture supernatants of HepG2 cells transfected with pHBV-1.2. HBV DNA levels were elevated to a lesser extent by IFIT3 silencing (Fig. 5B), whereas the overexpression of IFIT3 in HepG2 cells transfected with pHBV-1.2 markedly decreased HBsAg and HBeAg levels compared with those of the control (Fig. 5C). Consistently, the overexpression of IFIT3 had only a slight inhibitory effect on HBV replication (Fig. 5D). This observation was confirmed in one additional human hepatocyte cell line, namely, HL-7702. HL-7702 cells in which the endogenous expression of IFIT3 was specifically and stably knocked down by validated shRNAs were transfected with pHBV-1.2, and the levels of HBsAg, HBeAg, and HBV DNA secretion in the culture supernatants were elevated (Fig. 5E and F). In contrast, the overexpression of IFIT3 showed the opposite results (Fig. 5G and H). Thus, silencing the expression of IFIT3 could downregulate the anti-HBV effect of IFN-α.

## DISCUSSION

IFITs are a family of ISGs that play important roles in the antiviral process. Previous studies have reported that IFIT1 and IFIT2 are involved in the control of HBV by limiting initial HBV replication and spread (28). IFIT3 forms a more stable heterodimer with IFIT2 to associate with IFIT1, extends the half-life of IFIT1, and promotes the specificity of IFIT1 recognition for cap0 virus reporter mRNA, leading to an enhanced antiviral function of IFIT1 (29, 30). Yang et al. found previously that higher IFIT3 expression levels in liver tissues of patients with HCC predict better IFN-α therapeutic effects. Besides, the etiology of HCC patients included was related mostly to HBV infection (25). However, few studies have investigated the role and mechanism of IFIT3 in the progression of HBV infection (24). Since CHB patients can also be treated with IFN-α, we speculated that IFIT3 might be related to HBV infection. Investigating the role and mechanism of IFIT3 in the progression of HBV infection is of great significance.

Our study showed that IFIT3 was frequently upregulated in the peripheral blood of untreated patients with chronic HBV infection compared to healthy controls. Besides, IFIT3 was induced by HBV *in vitro*. The above-described results are consistent with the conclusion that IFIT3 belongs to the viral-stress-inducible genes (31), and IFIT3 transcriptional activation may be closely related to HBV infection. Following HBV exposure, liver damage is driven by the host immune response directed against virus-infected hepatocytes (32). It has been reported that HBeAg plays a key role in the host-virus interplay, particularly at the stage of viral antigen presentation and recognition by CD4^+^ T cells (33). During the process of clinical treatment, HBeAg clearance or HBeAg seroconversion can reflect viral control by the host, which is usually considered as one of the evidence of successful antiviral response (34) and/or reduced virus replication (34) and is believed to mark the end of immune-mediated liver damage (35, 36). Interestingly, patients who were HBeAg positive had significantly higher IFIT3 mRNA levels than did HBeAg-negative patients. Since greater viral adaptation was observed in HBeAg-negative individuals than in HBeAg-positive ones (37, 38), it is reasonable and logical to propose the association between IFIT3 and the host immune system in HBV replication. The relevance of IFIT3 for the antiviral effect of the host immune response needs to be examined in the future. The expression of IFIT3 was affected by chronic HBV infection; the overexpression of IFIT3 may be a common feature of patients with chronic HBV infection and might be used as a valuable biomarker similar to HBeAg or a target gene to promote a curative effect on patients.

It is well known that IFIT3 belongs to the ISGs, and the upregulation of IFIT3 expression is most evident after stimulation of Daudi cells by type I IFN (39). IFN induces the expression of ISGs, and the proteins encoded by these ISGs can inhibit different stages of virus replication (40). Some studies have shown that HBV behaves like a “stealth” virus and is not sensed by and does not actively interfere with the intrinsic innate immunity of infected hepatocytes, which is characterized by high quantities of viral particles and antigens in the circulation, with little or no type I and III IFNs in most HBV-infected patients (41–43). In contrast, several reports have challenged the notion of the stealth of the virus and suggested that HBV infection can activate the innate immunity of hepatocytes and trigger the production of type I or III IFNs (44, 45). Collectively, whether and how HBV can activate or block the production of IFN by liver cells remain controversial (41, 46, 47). Nonetheless, the therapeutic effect of IFN-α on the treatment of chronic HBV infection has been confirmed, and IFN-α has been widely applied. The present study showed that the expression of IFIT3 was increased significantly after the stimulation of both hepatocarcinoma cell lines and human hepatocytes by IFN-α, which is consistent with previous reports (28).

Previous studies demonstrated that phosphorylated/unphosphorylated STAT2 could form a complex with interferon regulatory factor 9 (IRF9), independent of STAT1, and bind to the IFN-stimulated gene regulatory element (ISRE) sequence on the IFIT3 promoter to promote IFIT3 transcription (48). Here, we identified IFIT3 as an ISG and found that STAT2 but not STAT1 was essential for the production of IFIT3. Moreover, the overexpression of IFIT3 increased the expression of PKR, OAS1, and MxA, classical antiviral ISGs induced by IFN-α that influence virus development through different mechanisms (49–51). Previous studies have found that STAT2 rather than STAT1 or STAT3 regulates dengue virus-induced IFIT3 production (22), which is similar to our results, suggesting that IFIT3 can play a role in a STAT2-dependent manner. In cells with the stable overexpression of IFIT3, IFIT3 played a role in the activation of the JAK-STAT signaling pathway through a STAT2-dependent mechanism and changed the expression of its downstream effector molecules; however, its specific antiviral mechanism warrants further exploration.

**FIG 6:**
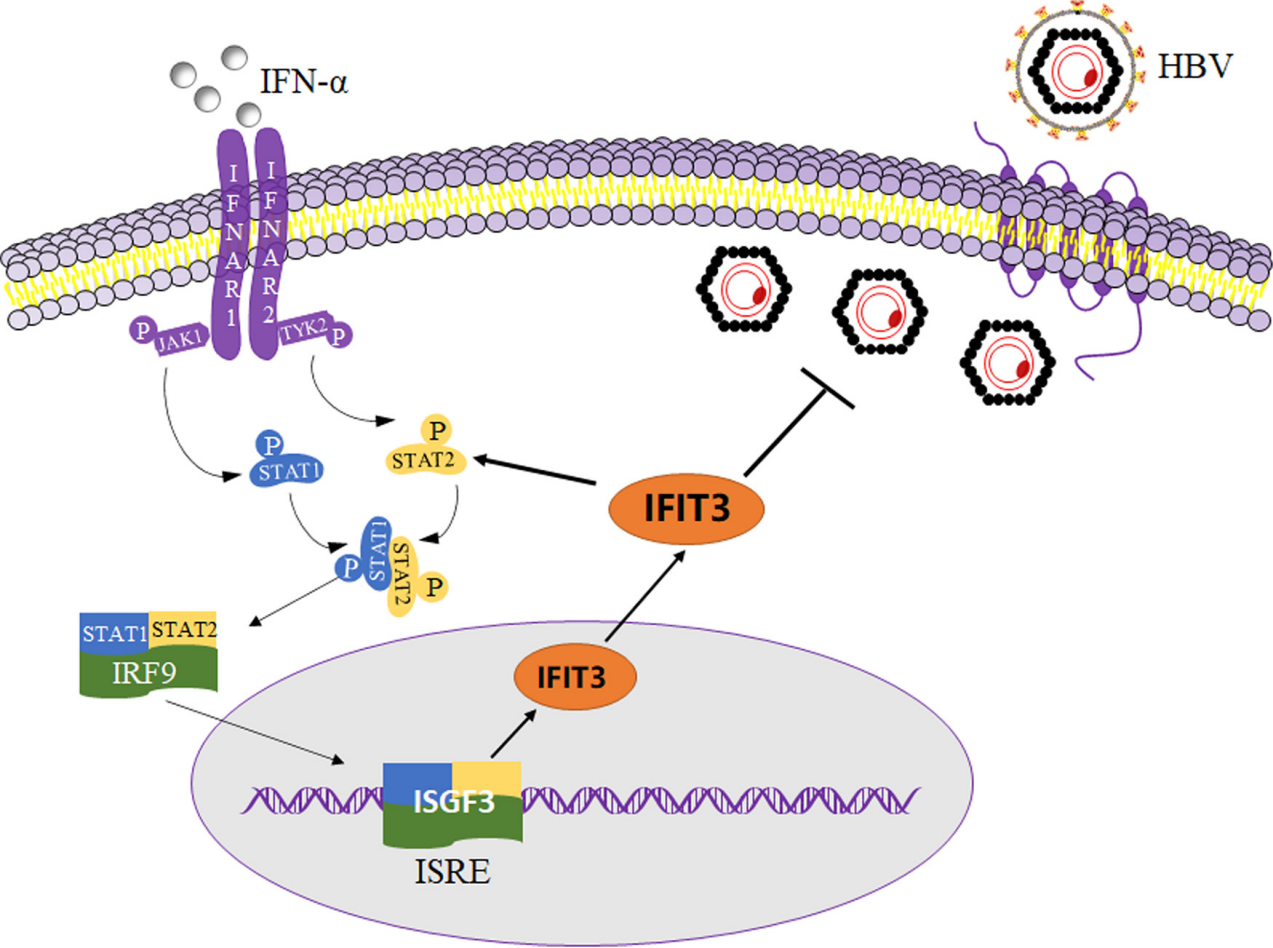
Working model for the IFIT3-mediated antiviral effect on hepatitis B virus (HBV) replication. The mechanism of IFIT3-mediated IFN-α antiviral steps after cells were infected with HBV is depicted. IFIT3 is shown as an oval shape, and its targeting sites on HBV are indicated by the bold solid line. NTCP, sodium taurocholate-cotransporting polypeptide; IFNAR, interferon alpha/beta receptor; JAK1/2, Janus-activated kinase 1/2; STAT1/2, signal transducer and activator of transcription 1/2; IRF9, interferon regulatory factor 9; ISGF3, interferon-stimulated gene factor 3 ISRE, interferon-stimulated gene regulatory element; ISG, interferon-stimulated gene; PKR, double-stranded RNA-activated protein kinase; MxA, myxovirus resistance A; OAS1, 2′-5′-oligoadenylate synthetase 1.

Generally, HBV can encode various proteins and be secreted from cells. As traditional serological markers associated with HBV, HBsAg, HBeAg, and HBV DNA can reflect the replication of HBV. We found that the inhibition of endogenous IFIT3 significantly increased the expression of HBV in HepG2 cells transfected with pHBV1.2 since the contents of HBsAg, HBeAg, and HBV DNA detected in the supernatants were significantly increased. The contents of HBsAg and HBeAg in the supernatants of cells were significantly lower in IFIT3-overexpressing HepG2 cells transfected with pHBV1.2 than in the control group. According to these results, IFIT3 affects HBV replication to an extent. As shown in Fig. 6, IFIT3 can be induced by targeting IFN-α. In addition, IFIT3 can play a positive-feedback role in regulating the antiviral function of IFN-α by affecting the phosphorylation of STAT2 *in vitro*.

### Conclusion.

In summary, our current study explored the mechanism of IFIT3 upregulation by HBV, whereas the overexpression of IFIT3 can inhibit HBV expression in both human hepatocytes and hepatocarcinoma cells, which contributed to revealing the pathogenesis of CHB. In addition, IFIT3 promotes the anti-HBV effect of IFN-α in a STAT2-dependent manner, providing the theoretical basis for the clinical treatment of CHB.

## MATERIALS AND METHODS

### Subjects.

A total of 178 participants were enrolled at the department of the Liver Disease Center of the First Affiliated Hospital of Fujian Medical University, including 70 healthy controls and 108 treatment-naive patients who were diagnosed with CHB according to *EASL 2017 Clinical Practice Guidelines on the Management of HBV Infection* (35). Patients were divided into four groups: hepatitis B e antigen (HBeAg)-positive patients with chronic hepatitis (group I), HBeAg-positive patients with chronic infection (group II), HBeAg-negative patients with chronic hepatitis (group III), and HBeAg-negative patients with chronic infection (group IV). All venous blood specimens were stored at −80°C until use. The baseline clinical characteristics of the study cohort are summarized in Table 1. This study was approved by the Ethics Committee of the First Affiliated Hospital of Fujian Medical University (approval no. MRCTA, ECFAH of FMU [2015]027). Written informed consent was obtained from all subjects before enrollment.

**TABLE 1:** Demographics of healthy controls and patient with chronic HBV infection^*a*^

Parameter	Value for chronic HBV infection group			*P* value for HBeAg^+^ vs HBeAg*^−^*^*b*^	Value for HCC group	*P* value for total chronic infection vs HCC^*c*^
	Total	HBeAg^+^	HBeAg*^−^*			
No. of patients	108	70	38		70	
No. of male/no. of female patients	57/51	34/36	23/15	0.23	44/26	0.18
Mean age (yrs) ± SD (range)	38.45 ± 10.49 (22–74)	35.41 ± 8.92 (22–57)	44.05 ± 10.96 (24–72)	<0.01	38.09 ± 10.29 (24–59)	0.82
Mean HBsAg concn (IU/mL)		41,970.88 ± 33,173.245	3,889.57 ± 5,788.40	<0.01		
Mean HBeAg concn ± SD (S/CO)		1,084.36 ± 542.93	0.45 ± 0.078	<0.01		
Mean HBV DNA concn (log_10_ IU/mL) ± SD	6.22 ± 1.94	7.22 ± 1.43	4.38 ± 1.32	<0.01		
Mean ALT concn (U/L) ± SD	70.49 ± 79.65	65.93 ± 76.27	78.03 ± 84.16	0.45	24.04 ± 17.01	<0.01
Mean AST concn (U/L) ± SD	45.57 ± 47.19	44.04 ± 47.22	48.39 ± 47.64	0.65	21.84 ± 7.91	<0.01
Mean TBIL concn (μmol/L) ± SD	13.79 ± 5.51	13.31 ± 5.83	14.68 ± 4.81	0.22	4.61 ± 0.73	<0.01
Mean DBIL concn (μmol/L) ± SD	4.45 ± 2.48	4.29 ± 2.79	4.75 ± 1.78	0.36	4.09 ± 1.366	0.35
Mean IBIL concn (μmol/L) ± SD	9.43 ± 3.63	9.16 ± 3.74	9.93 ± 3.41	0.30	9.67 ± 3.194	0.68
Mean GGT concn (U/L) ± SD	34.81 ± 33.23	30.2 ± 22.58	43.29 ± 46.12	0.11	27.09 ± 22.10	0.09
Mean TCHO concn (mmol/L) ± SD	4.83 ± 0.95	5.02 ± 1.03	4.54 ± 0.73	0.02	1.30 ± 0.74	<0.01
Mean TP concn (g/L) ± SD	72.7 ± 4.43	71.98 ± 4.61	74.06 ± 3.79	0.02	72.49 ± 4.33	0.78
Mean ALB concn (g/L) ± SD	43.70 ± 3.93	43.36 ± 4.07	44.35 ± 3.60	0.22	45.4 ± 2.51	<0.01
Mean GLO concn (g/L) ± SD	29.01 ± 3.25	28.62 ± 3.32	29.72 ± 3.03	0.10	27.30 ± 2.97	<0.01
Mean PLT (10^9^/L) ± SD	216.42 ± 60.817	214.18 ± 61.01	220.53 ± 61.10	0.62	261.13 ± 57.128	<0.01
Mean RDW (%) ± SD	13.53 ± 1.12	13.61 ± 1.23	13.39 ± 0.88	0.36	13.25 ± 0.78	0.07

a Fisher’s exact test and Student’s *t* test were used to compare categorical and continuous variables between the two groups, respectively. HC, healthy control; TCHO, total cholesterol; TP, total protein; ALB, albumin; GLO, globularproteins; PLT, platelet; RDW, red blood cell distribution width.

b Difference between the HBeAg^+^ and HBeAg*^−^* chronic HBV infection patient groups.

c Difference between the total chronic HBV infection patients and the HC group.

### Clinical chemistry analysis.

Serum total bilirubin (TBIL), direct bilirubin (DBIL), indirect bilirubin (IBIL), aspartate transaminase (ALT), glutamic-oxalacetic transaminase (AST), glutamyltranspeptidase (GGT), apolipoprotein B (APO-B), and low-density lipoprotein cholesterol (LDL-C) were measured using a cobas8000 automatic biochemical analyzer (Roche Diagnostics, Switzerland). Serum hepatitis B surface antigen (HBsAg), anti-HBs, HBeAg, anti-HBe, and anti-HBc were detected using the Architect ci4100 automatic biochemical immunoassay system (Abbott Laboratories, USA). Serum HBV DNA was quantified on the Roche LightCycler 480 system (Roche Corporation, Switzerland) using a quantitative real-time PCR (qRT-PCR) assay (Sansure Biotech, China). Complete blood counts were performed using the Advia 2120i automatic blood analyzer (Siemens, Germany).

### qRT-PCR analysis.

Purified RNA was reversely transcribed into cDNA using the RevertAid first-strand cDNA synthesis kit (catalog no. K1622; Thermo Fisher, USA). qRT-PCR analysis was performed with an ABI 7500 real-time PCR system (Life Technologies, USA) using TB Green premix Ex*Taq* (TaKaRa, Japan). All reactions were performed with the following conditions: 95°C for 30s, 40 cycles of 95°C for 10 s, and 60°C for 30 s. The specificity of the PCR products was ensured by melting curve analysis following each reaction. The relative expression of each mRNA was determined using the 2−ΔΔCt method with GAPDH as the endogenous control for data normalization. The primers used in this study are shown in Table S1 in the supplemental material.

### Cell culture and drugs.

HepG2 and HL-7702 cell lines were obtained from the Cell Bank of Type Culture Collection (Chinese Academy of Sciences, Shanghai, China). Cells were maintained in Dulbecco’s modified Eagle medium (DMEM; Gibco, USA) containing 10% fetal bovine serum (FBS; Gibco, USA) at 37°C in a 5% CO_2_ incubator. HepG2 and HL-7702 cells were transfected with lentivirus carrying the IFIT3 gene (Lv-IFIT3) /negative control (Lv-control) or viruses carrying small hairpin RNAs (shRNAs) targeting IFIT3 (sh-IFIT3#1 and sh-IFIT3#2)/negative control (sh-control) to obtain cell lines with the stable overexpression or knockdown of IFIT3. Cells were selected by puromycin to achieve a strong silencing/enhancing effect of shRNAs. All viruses were purchased from GeneChem (Shanghai, China). The infection efficiency was confirmed by qRT-PCR and Western blot assays. Cells were incubated in medium alone or medium with recombinant human IFN-α or phosphate-buffered saline (PBS) (Beyotime Biotechnology, China) for the indicated times (30 min, 6 h, 12 h, 24 h, or 48 h).

### Plasmids and antibodies.

The replication-competent HBV genome construct pREP-HBV1.2 (pHBV-1.2) was provided by Lin Xu. Plasmids were transfected into HepG2 or HL-7702 cells using Lipofectamine 3000 (Invitrogen, Thermo Fisher, USA) according to the manufacturer’s instructions. Anti-IFIT3 antibody (catalog no. ab236243) was purchased from Abcam (USA); anti-STAT2/phospho-STAT2, anti-Mx1, anti-PKR, anti-OAS1, and anti-glyceraldehyde-3-phosphate dehydrogenase (GAPDH) antibodies were obtained from Cell Signaling Technologies (USA); and horseradish peroxidase (HRP)-conjugated anti-rabbit IgG and HRP-conjugated anti-mouse IgG antibodies were purchased from Beyotime Biotechnology (China).

### Western blot assays.

Cells were washed twice with ice-cold PBS, lysed with 1× radioimmunoprecipitation assay (RIPA) buffer (Beyotime Biotechnology, China), kept on ice for 30 min, and then centrifuged at 13,000 rpm for 15 min. After protein estimation, sodium dodecyl sulfate (SDS) loading dye was added to the samples, and the samples were boiled at 100°C for 10 min. Clarified lysates were resolved by SDS-polyacrylamide gel electrophoresis (PAGE) and transferred to polyvinylidene difluoride (PVDF) membranes (Beyotime Biotechnology, China). The membranes were blocked with 5% nonfat milk for 1 h and incubated overnight at 4°C with primary antibodies (1:1,000) diluted in 2% bovine serum albumin. After washing, the membranes were incubated with HRP-conjugated secondary antibodies (1:5,000) for 1 h. Signals were detected using a ChemiDoc XRS^+^ chemiluminescence imaging system (Bio-Rad, USA).

### Statistical analysis.

Data sets were downloaded from the Gene Expression Omnibus (GEO) database (https://www.ncbi.nlm.nih.gov/geo/) (accession no. GSE118295 and GSE69590) as MINIML files. Box plots were drawn using the R software package ggplot2.

Fisher’s exact test and Student’s *t* test were used to compare categorical and continuous variables between two groups, respectively. Normally distributed variables are presented as means ± standard deviations (SD) and were analyzed by two-tailed Student’s *t* test or one-way analysis of variance (ANOVA). Besides, for nonparametric data, the Mann-Whitney U test was used for comparisons between groups. All statistical analyses were carried out using SPSS22.0 software (SPSS Inc., USA) and GraphPad Prism 7.0 software (GraphPad Software Inc., USA). A two-tailed *P* value of <0.05 was considered statistically significant.
