# Supplementary material for: IFIT3 Is Increased in Serum from Patients with Chronic Hepatitis B Virus (HBV) Infection and Promotes the Anti-HBV Effect of Interferon Alpha via JAK-STAT2 In Vitro
Source: Microbiol Spectr. 2022 Oct 31;10(6):e01557-22. doi: 10.1128/spectrum.01557-22 (PMC9769971; doi:10.1128/spectrum.01557-22)
Supplement: Supplemental file 1 — Table S1 and Fig. S1. Download spectrum.01557-22-s0001.pdf, PDF file, 0.2 MB [file spectrum.01557-22-s0001.pdf]

Table S1

Primer sequence of target gene (human source)

Gene name	primer	Primer sequence（5’-3’）
GAPDH	FW	GGAGCGAGATCCCTCCAAAAT
	RV	GGCTGTTGTCATACTTCTCATGG
IFIT3	FW	ATGGTAGTGAGGAAATGGGC
	RV	TGTCTTCCTATCGTCCTACCC
MXA	FW	GCATCCCACCCTCTATTACTG
	RV	AGTCAATGAGGTCGATGCAG
OAS1	FW	TGAGAAGGCAGCTCACGAAA
	RV	CATCGTCTGCACTGTTGCTT
PKR	FW	CGATACATGAGCCCAGAACAG
	RV	AGAATTAGCCCCAAAGCGTAG

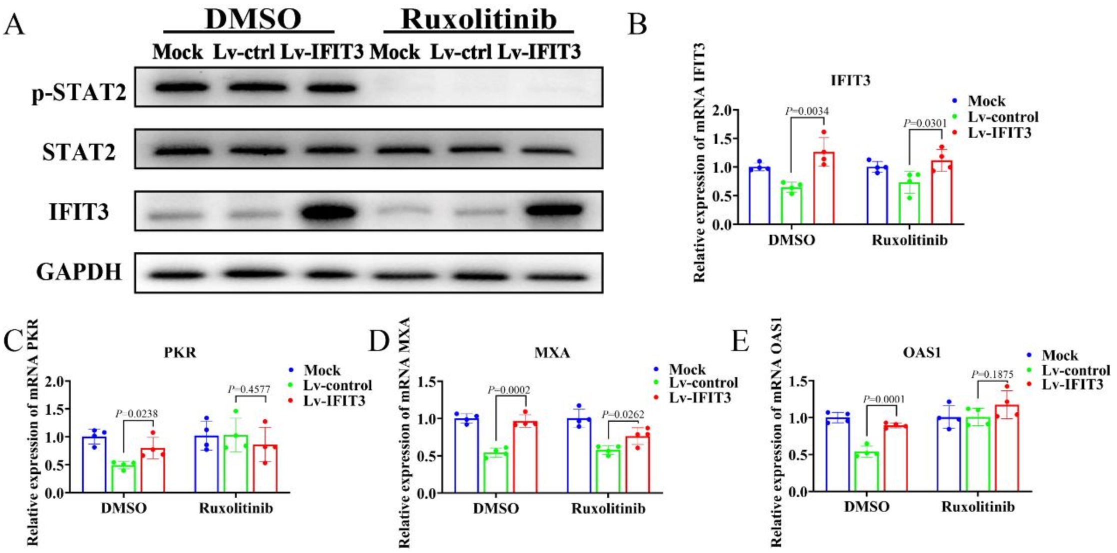

Figure S1. Inhibition the phosphorylation of STAT2 weaken the role of IFIT3 in strengthening IFN-α effect

(A) HL-7702 cells with enhanced IFIT3 were pre-treated with Ruxolitinib for 6 h before treatment with IFN-α (1,000 IU/ml) for 30 min. Cells were treated with dimethyl sulfoxide (DMSO), which served as a negative control. (B-E) HL-7702 cells with enhanced IFIT3 were pre-treated with Ruxolitinib/DMSO for 6 h, and then cultured with IFN-α (1,000 IU/ml) for 12 h. A control (without Ruxolitinib/DMSO) was used in each treatment group. The experiments were performed in triplicate and data were expressed as mean± SD.
